# An Enhanced Snow Geese Optimizer Integrating Multiple Strategies for Numerical Optimization

**DOI:** 10.3390/biomimetics10060388

**Published:** 2025-06-11

**Authors:** Baoqi Zhao, Yu Fang, Tianyi Chen

**Affiliations:** 1 Institute of Artificial Intelligence Application, Ningbo Polytechnic, Ningbo 315800, China; 2021012@nbpt.edu.cn; 2 School of Artificial Intelligence, Ningbo Polytechnic, Ningbo 315800, China; 3 Taizhou Institute of Zhejiang University, Taizhou 318000, China; 4 Solar Energy Research Institute of Singapore, National University of Singapore, Singapore 117574, Singapore; e0348767@u.nus.edu

**Keywords:** snow geese algorithm, meta-heuristic algorithm, adaptive switching strategy, dominant group guidance, dominant stochastic difference search, CEC 2017 and CEC 2022 benchmark functions, robot path planning

## Abstract

An enhanced snow geese algorithm (ESGA) is proposed to address the problems of the weakened population diversity and unbalanced search tendencies encountered by the snow geese algorithm (SGA) in the search process. First, an adaptive switching strategy is used to dynamically select the search strategy to balance the exploitation and exploration capabilities. Second, a dominant group guidance strategy is introduced to improve the population quality. Finally, a dominant stochastic difference search strategy is designed to enrich the population diversity and help it escape from the local optimum by co-directing effects in multiple directions. Ablation experiments were performed on the CEC2017 test set to illustrate the improvement mechanism and the degree of compatibility of their improved strategies. The proposed ESGA with a highly cited algorithm and the powerful improved algorithm are compared on the CEC2022 test suite, and the experimental results confirm that the ESGA outperforms the compared algorithms. Finally, the ability of the ESGA to solve complex problems is further highlighted by solving the robot path planning problem.

## 1. Introduction

### 1.1. Research Background

The effectiveness of deterministic algorithms has been greatly limited in the past few years because of the growing intricacy of optimization problems in real-world situations. Conversely, as a stochastic algorithm, meta-heuristic (MH) algorithms have demonstrated remarkable efficacy in addressing these issues. MH algorithms are used in solving optimization problems, first in the problem space to generate a number of random solutions, and then in the space with a certain search rule to adjust the location of these solutions, use evaluation mechanisms to consider the individual advantages and disadvantages, and use this to adjust the direction of the final optimal solution in order to lock [1]. MH algorithms have been extensively utilized in diverse domains such as path planning [2,3], feature selection [4,5], and image segmentation [6,7]. In consideration of the no free lunch (NFL) theorem [8], which states that there is no one algorithm that can consistently outperform all optimization problems, it is essential to explore meta-heuristic (MH) algorithms, as no individual algorithm can attain optimal performance across all optimization problems.

### 1.2. Contribution of the Work

This work proposes an enhanced snow geese algorithm (ESGA), which improves the basic SGA through the adaptive switching strategy, dominant group guidance strategy, and dominant stochastic difference search strategy. The performance and potential of the proposed ESGA are comprehensively examined on the CEC2017 test suite, the CEC2022 test suite, and the robot path planning problem. The results show that the ESGA is a promising SGA variant.

### 1.3. Section Arrangement

A literature review is conducted in Section 2. Section 3 provides an overview of the SGA. Section 4 details the proposed ESGA. Section 5 presents the benchmark test function experiments and robot path planning trials. Finally, the conclusions are drawn in Section 6.

## 2. Literature Review

Among the many meta-heuristic algorithms, the sources of inspiration are varied and unique. Most of these algorithms draw on the essence of various types of behaviors of creatures or animals in nature. Among these algorithms, the Genetic Algorithm (GA) [9], Particle Swarm Optimization (PSO) [10], and Simulated Annealing (SA) [11] are the three most well-known algorithms. GA is an evolution-based algorithm inspired by the principles of Darwinian-based evolution and Mendelian genetics. It progressively optimizes the problem solution by simulating natural selection and genetic mechanisms. PSO is a swarm-based meta-heuristic algorithm inspired by the foraging behavior of bird flocks. Its basic idea is to optimize the solution of the problem step by step by simulating the information sharing and collaboration between individuals of a flock of birds during the foraging process. SA is a physics-based algorithm derived from the annealing process of solid materials. SA finds the global optimal solution by gradually decreasing the temperature of the system. With the development of meta-heuristic algorithms, researchers continue to present more powerful and novel algorithms. Tuna Swarm Optimization (TSO) [12] originated from two behavioral patterns of spiral and parabolic foraging in tuna. The Secretary Bird Optimization Algorithm (SBOA) originated from the behavioral patterns of secretary birds constantly searching for prey and evading predators [13]. The Spider Wasp Optimizer (SWO) replicates the foraging, nest construction, and reproductive activities of female spider wasps [14]. Dwarf Mongoose Optimization (DMO) draws inspiration from the foraging behaviors exhibited by dwarf mongeese, which exert a profound influence on the social dynamics and ecological adaptability of these species [15]. The Sea Horse Optimizer (SHO) draws inspiration from the natural actions of sea horses, including their patterns of movement, methods of foraging, and habits related to reproduction [16]. Inspired by the natural behavior of crayfish, the Crayfish Optimization Algorithm (COA) [17] solves complex optimization problems by simulating their mechanisms of temperature adaptation, burrow selection, and foraging competition. The Polar Lights Optimizer (PLO) [18] is a meta-heuristic algorithm based on the phenomenon of polar lights to efficiently solve various complex optimization problems by simulating the rotational motion of charged particles in the Earth’s magnetic field, their walks in the auroral ellipse region, and inter-particle collisions. Henry Gas Solubility Optimization (HGSO) [19] is a physics-based meta-heuristic algorithm inspired by Henry’s law, which describes the solubility of a gas in a liquid as proportional to the partial pressure of that gas. The HGSO algorithm exploits and explores the search space in a balanced manner by modeling the aggregation behavior of gases.

The snow geese algorithm (SGA) is a swarm-based meta-heuristic algorithm proposed by Tian et al. in 2024 [20]. The SGA is inspired by the migratory behavior of snow geese, in particular the unique “herringbone” and “straight line” flight patterns that they form during migration. The algorithm achieves efficient search and optimization in the solution space by simulating the flight behavior of snow geese. Wu et al. developed an agent-assisted multi-objective snow geese algorithm based on Gaussian processes and optimized the cold chain logistics problem [21]. Chandrashekhar et al. applied the SGA to optimize the performance of on-board chargers for electric vehicles [22]. Although the SGA achieves better performance by virtue of its unique search method, it still faces the disadvantage of insufficient population diversity when dealing with complex situations, and at the same time, it is difficult for it to balance exploitation and exploration, hence it falling into the local optimum. Bian et al. comprehensively strengthened the search capability of the SGA by proposing a lead-geese rotation mechanism, a horn-oriented mechanism, and an outlier boundary strategy [23]. In order to better utilize the capability of the SGA, this paper proposes an ESGA approach that integrates three improvement strategies. First, an adaptive switching strategy is used to dynamically select the search strategy to balance the exploitation and exploration capabilities. Second, a dominant group guidance strategy is introduced to improve the population quality. Finally, a dominant stochastic difference search strategy is designed to enrich population diversity and help it escape from the local optimum by co-directing effects in multiple directions.

## 3. The Basic SGA

### 3.1. Inspiration Source

The snow geese algorithm, proposed by Tian et al. in 2024 [20], is a swarm-based meta-heuristic algorithm. The algorithm was conceived based on the unique formation structure of snow geese during migration and the corresponding change patterns of this formation structure under different circumstances. The SGA consists of three phases: the initialization phase, exploration phase, and exploitation phase. The critical operations of this algorithm, along with their corresponding mathematical models, are presented below.

### 3.2. Initialization Phase

In the SGA, each member of the population represents a solution. Each solution consists of D elements that fulfill the restrictions of the boundary conditions. These solutions then collectively form the population. Like other meta-heuristic algorithms, the first step of the SGA is to generate the initial population. Assuming that the search range of the problem space is $\left[lb,ub\right]$, the position of the $i^{th}$ solution, $P_{i}$, can be given by Equation (1):

(1) $$P_{i}=lb+rand\left(1,D\right)\times \left(ub-lb\right),i=1,2,\ldots ,N$$

In Equation (1), $rand\left(1,D\right)$ is a D dimensional random vector whose elements are random numbers ranging from 0 to 1. N is the number of SGA population members. After obtaining the initial population, we will evaluate the fitness of each individual, denoted as Equation (2).

(2) $$fit_{i}=F\left(P_{i}\right)$$

where $F\left(\cdot \right)$ denotes the objective function. $fit_{i}$ represents the fitness of the $i^{th}$ member.

### 3.3. Exploration Phase

The SGA achieves extensive searching during the exploration phase by modeling the migration process of the herringbone formation of the snow geese population. In this phase, the SGA divides the population into three parts, corresponding to different updating methods, as follows. The populations are divided according to the magnitude of fitness. For individuals ranked in the top twenty percent of fitness, the SGA updates their position using Equation (2).

(3) $$P_{i}^{t+1}=P_{i}^{t}+a\times \left(P_{best}^{t}-P_{i}^{t}\right)+V_{i}^{t}$$

(4) $$V_{i}^{t+1}=\frac{4V_{i}^{t}\times t}{T\times e^{\frac{4t}{T}}}+P_{best}^{t}-P_{i}^{t}-0.005\times 1.29\times {\left(V_{i}^{t}\right)}^{2}\times \sin\left(\theta \right)$$

where $P_{i}^{t}$ represents the position of the $i^{th}$ agent at iteration t. $P_{best}^{t}$ is the position of the best agent (with best fitness value) at iteration t. T denotes the total number of allowable iterations. The constants in Equation (4) are taken from the original literature. For those individuals ranked in the middle of the fitness scale, the SGA updates their position using Equation (5).

(5) $$P_{i}^{t+1}=P_{i}^{t}+a\times \left(P_{best}^{t}-P_{i}^{t}\right)+b\times \left(P_{c}^{t}-P_{i}^{t}\right)-d\times \left(P_{worst}^{t}-P_{i}^{t}\right)+V_{i}^{t}$$

(6) $$P_{c}^{t}=\frac{\sum_{i=1}^{N}P_{i}^{t}\times fit_{i}}{N\times \sum_{i=1}^{N}fit_{i}}$$

For the remaining individuals (fitness ranked in the bottom twenty percent), the SGA applies Equation (7) to update their positions.

(7) $$P_{i}^{t+1}=P_{i}^{t}+a\times \left(P_{best}^{t}-P_{i}^{t}\right)-b\times \left(P_{c}^{t}-P_{i}^{t}\right)+V_{i}^{t}$$

where $P_{worst}^{t}$ represents the position of the worst agent at iteration t. The a,b,d appearing in Equation (3), Equation (5), and Equation (7) are empirical values obtained experimentally as follows:

(8) a=4×rand−2

(9) b=3×rand−1.5

(10) d=2×rand−1

### 3.4. Exploitation Phase

The SGA can switch between exploitation and exploration behaviors via angle θ. When θ<π, the SGA uses a linear flight mode that contains the following two mechanisms:

(11) $$P_{i}^{t+1}=P_{i}^{t}-\left(P_{best}^{t}-P_{i}^{t}\right)\times rand,rand>0.5$$

(12) $$P_{i}^{t+1}=P_{i}^{t}-\left(P_{best}^{t}-P_{i}^{t}\right)\times rand\times Brownian\left(D\right),rand\leq 0.5$$

(13) $$\theta =\frac{2\pi t}{T}$$

where $Brownian\left(D\right)$ are the values of standard Brownian motion that obey a normal distribution with mean 0 and standard deviation 1. rand is a random number ranging from 0 to 1.

## 4. The Proposed ESGA

The performance of the SGA decreases significantly when facing complex optimization problems. This is due to the fact that it neglects the guiding role of the dominant population, which makes it difficult to improve the quality of the population. It is difficult to maintain a coordinated search with a single exploitation or exploration behavior. Utilizing only the information of the optimal individuals in the exploitation stage easily leads to the SGA falling into local optimality. Therefore, this paper proposes three improvement strategies: the adaptive switching strategy, dominant group guidance strategy, and dominant stochastic difference search strategy to address these drawbacks encountered by the SGA.

### 4.1. Adaptive Switching Strategy (ASS)

**Figure 1:**
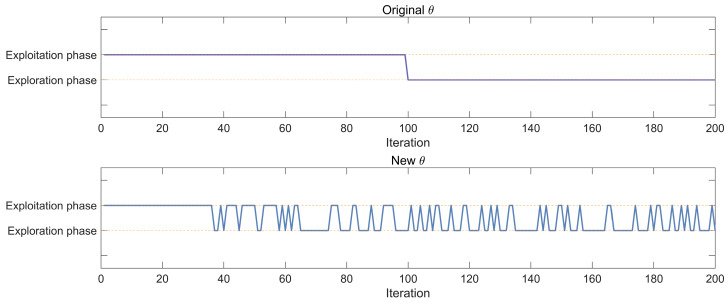
The value of original θ and new θ.

In general, meta-heuristic algorithms require searching for more unknown regions upfront and selecting promising regions for deeper exploitation at a later stage. The switching mechanism of the SGA does exactly that. However, the switching mechanism of the SGA is fixed-switching, i.e., only global exploration in the early stage and only local exploitation in the later stage. This search mechanism, which only performs a single exploitation or exploration in any period, limits the performance of the algorithm. In this paper, we propose an adaptive switching strategy to overcome this shortcoming. As shown in Figure 1, the strategy performs a more global search in the early stage, while retaining some local exploitation capability, which can improve the convergence speed of the algorithm. In the later stage, the strategy ensures the better execution of the exploitation strategy and also has the ability of global exploration, which can enrich the diversity of the population and avoid falling into the local optimum. It is calculated that during the first half of the iterations, the exploration and exploitation are 75% and 25%, respectively, and during the second half of the iterations, these percentages are reversed. The modified version of θ is updated in the following way:

(14) $$\theta =2\pi \times \sin\left(\left(\frac{\pi t}{T}\right)\times rand\right)$$

### 4.2. Dominant Group Guidance Strategy (DGS)

During the global exploration phase, the most numerous populations in the SGA update their position through Equation (5). This updating method utilizes the positions of the best individual, the worst individual, and the average position of the entire population for guidance. Although it is able to expand the search to some extent, this approach may lead to a stagnant search. This is because the guidance of the best and worst individuals may be constrained by each other, while the average position of the whole population cannot guide promising directions. Based on the above analysis, this work proposes a dominant group guidance strategy (DGS). The DGS fits the distribution of the dominant group through a Gaussian probability distribution model, which in turn yields the evolutionary trend of the population. This method utilizes the guidance of dominant groups, which can enhance the quality of the population and promote the search. In addition, we use the position of each individual to modify the evolutionary trend, which can enrich the population diversity. The DGS can be expressed in the following equation:

(15) $$P_{i}^{t+1}=P_{wmean}^{t}+P_{i}^{t}+g,g∼N\left(0,Cov\right)$$

(16) $$P_{wmean}^{t}=\sum_{i=1}^{0.5N}\left(\ln\left(0.5N+1\right)/\left(\sum_{i=1}^{0.5N}\left(\ln\left(0.5N+1\right)-\ln\left(i\right)\right)\right)\right)\times P_{i}^{t}$$

(17) $$Cov=\frac{1}{0.5N}\times \sum_{i=1}^{0.5N}\left(P_{i}^{t}-P_{wmean}^{t}\right)\times {\left(P_{i}^{t}-P_{wmean}^{t}\right)}^{T}$$

where $P_{wmean}^{t}$ is the weighted average position of the top half of the populations ranked in terms of fitness. $P_{wmean}^{t}$ is used instead of $P_{c}^{t}$, which better reflects the search direction of the whole population. At the same time, different positions have different degrees of influence on the search direction, and the better the individual, the greater its influence on $P_{wmean}^{t}$.

### 4.3. Dominant Stochastic Difference Search Strategy (DSS)

During the exploitation phase, the SGA performs an in-depth search for promising regions in two ways. This approach of Equation (11) allows each individual to move closer to the optimal solution, which accelerates convergence, but at the same time diminishes population diversity while risking falling into a local optimum. In general, when the optimal individual falls into local optimality, the rest of the individuals approaching it are also prone to falling into local optimality. To address this problem, a dominant stochastic difference search strategy is proposed in this paper, which introduces the bootstrapping of three factors, including the bootstrapping of the dominant group, the bootstrapping of the stochastic individuals, and the bootstrapping of the historical individuals, as shown in Equation (18).

(18) $$P_{i}^{t+1}=P_{i}^{t}+\left(P_{pbest}^{t}-P_{i}^{t}\right)\times rand+\left(P_{r1}^{t}-P_{A}^{t}\right)\times rand,rand>0.5$$

where $P_{pbest}^{t}$ is a randomly selected individual from the top ten percent of individuals in terms of fitness. $P_{r1}^{t}$ denotes a randomly selected individual from the entire population. $P_{A}^{t}$ is a randomly selected individual from a set A. This set A contains historical information about each individual, and current population information, which can enhance population diversity.

### 4.4. The Framework of the ESGA

**Figure 2:**
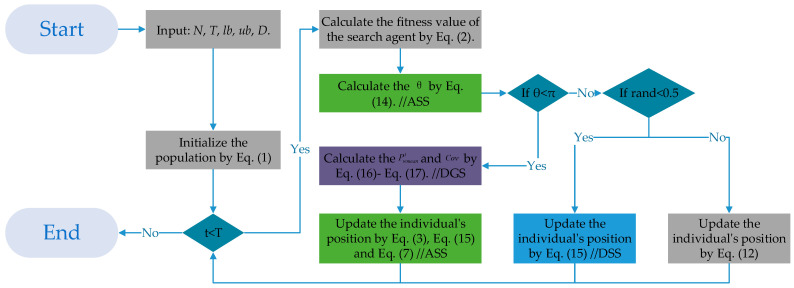
The flowchart of ESGA.

This subsection introduces the framework of the ESGA. The pseudo-code of the proposed ESGA is exhibited in Algorithm 1. The flowchart of the ESGA combining the adaptive switching strategy, dominant group guidance strategy, and dominant stochastic difference search strategy is shown in Figure 2.
**Algorithm 1: Pseudo-Code of ESGA**Input: *N*, *T*, *lb*, *ub*, *D*. Initialize the population using Equation (1). FOR *t* = 1: *T*     Calculate the fitness value of the search agent using Equation (2).     Calculate the *θ* using Equation (14). //ASS     IF θ<π     Calculate the $P_{wmean}^{t}$ and Cov using Equations (16) and (17). //DGS          Update the individual’s position using Equations (3), (7), and (15).     ELSE          IF rand < 0.5               Update the individual’s position using Equation (18). //DSS          ELSE               Update the individual’s position using Equation (12).          END IF     END IF END FOR Output: The best position and the best fitness.

### 4.5. Complexity Analysis of the ESGA

Time complexity is essential for assessing an algorithm’s performance efficiency. In swarm-based optimization algorithms, time complexity mainly depends on factors such as population size, dimensionality, the number of iterations, and the computational cost of the fitness function. The time complexities of the SGA and ESGA are as follows.

In the SGA, the initialization function generates *N* individuals, each with a dimension of *D*. Therefore, the time complexity of this step is $O\left(N\times D\right)$. The complexity of each fitness computation is $O\left(N\right)$. In the position update operation, the outer loop runs N times, while the inner loop iterates D times. Therefore, the time complexity of this part is $O\left(N\times D\right)$. Given that the algorithm runs for T iterations, the time complexity of the SGA is $O\left(N\times D\times T\right)$.

In the ESGA, the time complexity of the initialization function and the computation of the fitness value are unchanged and remain $O\left(N\times D\right)$ and $O\left(N\right)$, respectively. In the position update operation, the outer loop runs N times, while the inner loop iterates D times. Therefore, the time complexity of this part is $O\left(N\times D\right)$. The ASS is used to replace the original update method of θ without increasing the time complexity. The DGS and DSS are used to replace the original search strategy without increasing the time complexity. Given that the algorithm runs for T iterations, the time complexity of the ESGA is $O\left(N\times D\times T\right)$. Combining the above complexities, the time complexity of the ESGA is of the same order of magnitude as that of the SGA, and its performance improvements are not achieved through increased complexity.

## 5. Experimental Results and Analysis

The performance of the proposed ESGA was evaluated using the CEC2017 and CEC2022 benchmark functions to validate its performance. The results were compared with six other meta-heuristic algorithms: MRFO [24], DBO [25], EO [26], RIME [27], GLS-MPA [28], EOSMA [29], AFDB-ARO [30], and LSHADE [31]. These four basic algorithms are widely used algorithms. The four improved algorithms have proven their excellent performance in the respective literature on them. The convergence curves of each algorithm across each benchmark function were analyzed, illustrating the convergence speed, stability, and precision of the optimization process for each function. Additionally, the mean value, standard deviation, and optimal value for each algorithm on each benchmark function were examined, providing a comprehensive view of overall convergence performance, performance consistency, reliability under challenging conditions, and typical performance less sensitive to outliers. The significant difference between the ESGA and the competing algorithms was verified using the nonparametric Wilcoxon rank sum test and Friedman test.

The simulations and analyses for this experiment were conducted using MATLAB2023b on a Core i9-13900H processor with 32 GB of RAM running at 2.6 GHz. Table 1 provides the simulation parameters for each algorithm.

### 5.1. Ablation Experiments Using the CEC2017 Test Set

To examine the effectiveness of each improvement strategy, we tested the SGA variants integrating a single improvement strategy and two improvement strategies on the CEC2017 test suite. The details of the SGA variants are shown in Table 2, where “Y” denotes the integration of the strategy and “N“ indicates that the strategy is not included. Specifically, these include SGA-AS (the improved SGA with the adaptive switching strategy), SGA-DG (the improved SGA with the dominant group guidance strategy), SGA-DS (the improved SGA with the dominant stochastic difference search strategy), SGA-ASDG (the improved SGA with the adaptive switching strategy and dominant group guidance strategy), SGA-ASDS (the improved SGA with the adaptive switching strategy and dominant stochastic difference search strategy), and SGA-DGDS (the improved SGA with the dominant group guidance strategy and dominant stochastic difference search strategy). The benchmark functions of CEC2017 are divided into three categories: unimodal functions F1–F2, multimodal functions F3–F9, and hybrid and composite functions F10–F29. To ensure a more accurate comparison between these SGA variants, 30 independent runs are conducted for each algorithm in this experiment. In each run, the population size is initialized to 30, the dimensionality is set to 10/30/50/100, and the maximum number of function evaluations is set to 30,000, with the upper limit and the lower limit to be [−100, 100].

**Figure 3:**
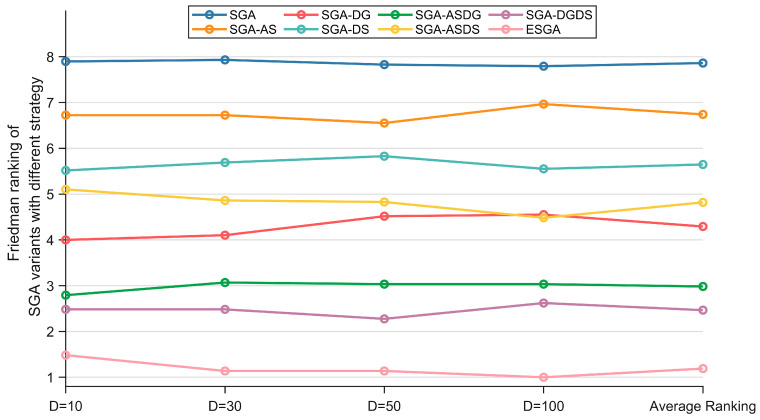
The Friedman ranking of SGA variants with different strategies on CEC2017 test suite.

Table 3 summarizes the results of the Friedman test based on the performance of ESGA, SGA, SGA-AS, SGA-DG, SGA-DS, SGA-ASDG, SGA-ASDS, and SGA-DGDS on the CEC2017 test suite, and the Friedman ranking is depicted as Figure 3. The algorithm data with good performance are shown in bold. Based on the p-values in the last column of Table 3, we can first conclude that there are significant differences between these algorithms. Then, in terms of the overall ranking, the ESGA containing all the improved strategies shows the best performance in solving the different dimensions of the problem, achieving an average ranking of 1.190. By comparing the SGA with the three SG variants combining a single improvement strategy, we can learn that all three improvement strategies improve the performance of the SGA in descending order of performance improvement: DGS, DSS, and ASS. By comparing the SGA variant incorporating two improvement strategies with the SGA variant incorporating a single improvement strategy separately, we can learn that the three strategies can mutually reinforce each other to further improve the SGA performance.

**Figure 4:**
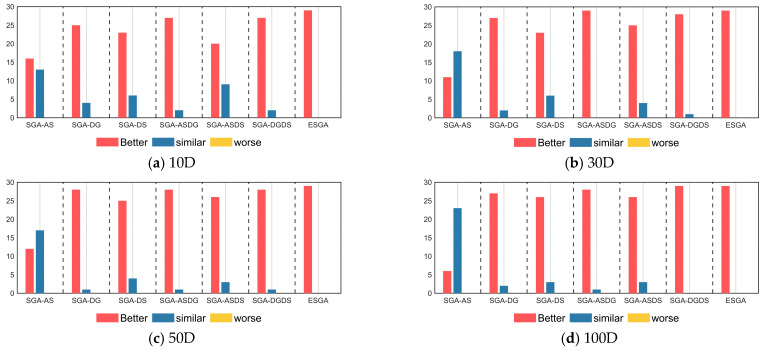
The Wilcoxon rank sum test results of SGA variants with different strategies on CEC2017 test suite.

Figure 4 presents the results of the Wilcoxon rank sum test between the SGA variant and the SGA. In Figure 4, “better” indicates that the SGA variant significantly outperforms the original SGA. Furthermore, “similar” indicates that the SGA variant and the original SGA have similar performance, while “worst” indicates that the SGA variant is significantly inferior to the original SGA. According to Figure 4, the ESGA significantly outperforms the original SGA in all functions for all four cases, which again illustrates the great strength of the ESGA. By observing the comparison between the SGA variants combining a single or two improvement strategies and the original SGA, we can conclude that all the improvement strategies will not significantly weaken the search capability of the original SGA, even though they do not enhance its performance. This demonstrates the effectiveness of the three improvement strategies proposed in this paper.

### 5.2. Comparison Test Using the CEC2022 Test Suite

**Figure 5:**
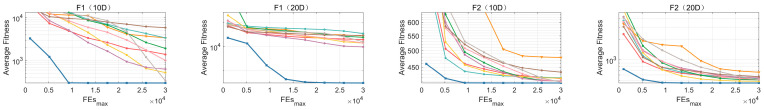

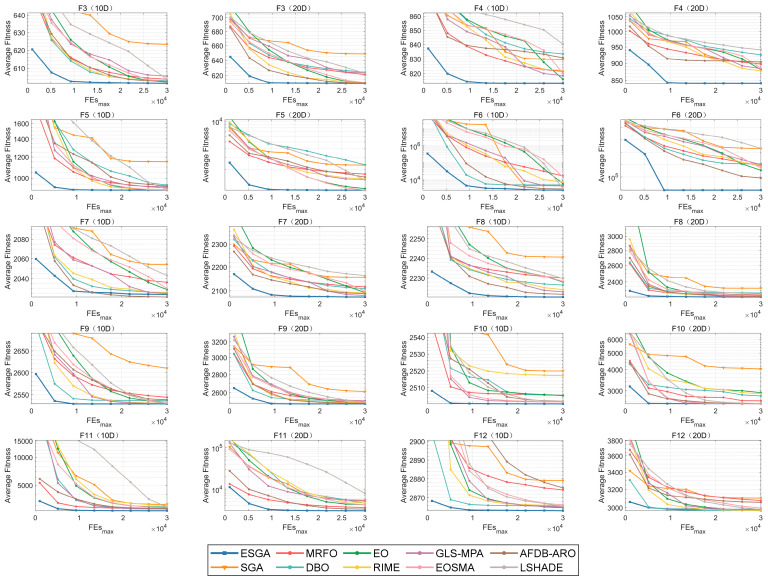
The convergence curves of ESGA and competitors on CEC2022 test suite.

**Figure 6:**
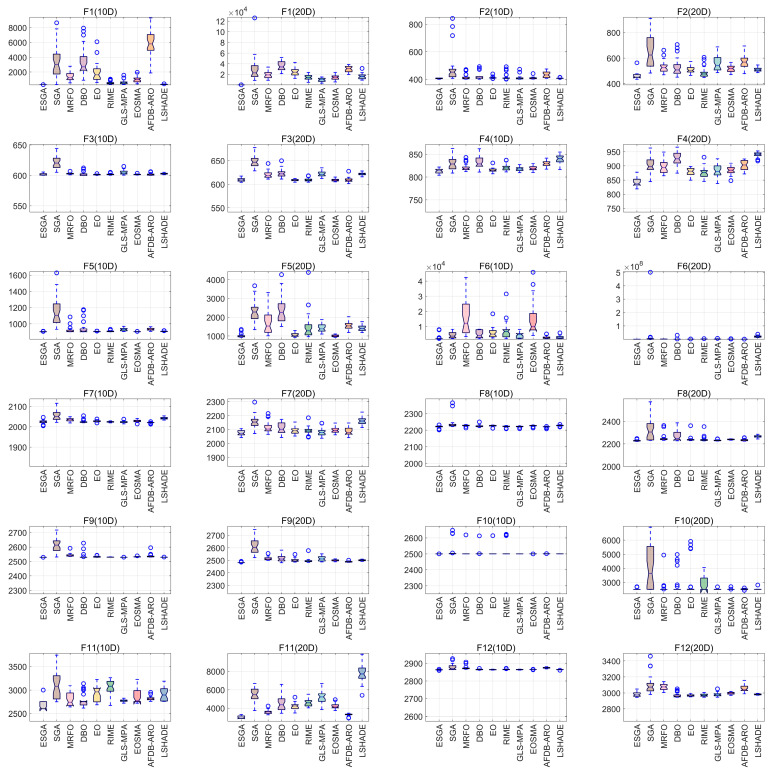
The boxplot curves of ESGA and competitors on CEC2022 test suite.

In this subsection, we further evaluate the performance of the ESGA by comparing it with MRFO, DBO, EO, RIME, GLS-MPA, EOSMA, AFDB-ARO, and LSHADE in the CEC2022 test set. The benchmark functions of CEC2022 are divided into three categories: unimodal functions F1, basic functions F2–F5, and hybrid and composite functions F6–F12. In order to be fair, we set the number of populations as 300, the dimensionality to 10/20, and the maximum number of function evaluations to 30,000, with the upper and lower limits as [−100, 100]. Each algorithm is run for 30 times, and the best value (Min), average value (Avg), and standard deviation (Std) of each algorithm are calculated. These three indicators can reflect the algorithm’s ability to find the best; in order to find out more clearly which algorithm has a clear advantage, we choose to put the data with the best average in bold. The experimental results are shown in Table 4 and Table 5, and the convergence curves and boxplots are shown in Figure 5 and Figure 6.

For the unimodal function F1, the ESGA achieves excellent performance on both 10D and 20D, obtaining the best average value, which indicates that the improvement strategy has enhanced the local search capability of the SGA to find the optimal solution more accurately. Combined with the standard deviation, the ESGA shows some stability on the unimodal functions. For the basic functions F2–F5, the ESGA shows the best performance when facing F2, F4, and F5 on 10D, and provides the best solution when solving F2 and F4 on 20D. Although the ESGA’s performance is not the best in F3, it still outperforms the original SGA. EO outperforms other algorithms on F3 on both 10D and 20D. EOSMA on F5 on 20D ranked first. Hybrid and combined functions featuring multiple peaks or valleys, multiple local optima, and a single global optimum are used to evaluate the global search capability and ability of the algorithm to get rid of local optima. When facing the 10D function, the ESGA can provide the best averages on F6 and F8–F12, weaker than GLS-MPA and AFDB-ARO only on F7, still outperforming the original SGA. For the 20D hybrid and combined functions, the ESGA outperforms the other algorithms in terms of averages on F6–F9 and F11. However, the ESGA does not perform as well on two functions, F10 and F12, ranking only fifth and sixth, respectively. Overall, in terms of the number of average values, the ESGA has more average values than the other algorithms, reflecting the robustness and adaptability of the improved ESGA in complex environments. From the data of minimum values, most of the values of the ESGA are smaller than those of the SGA and other comparative algorithms, indicating that the local search ability of the ESGA is enhanced and the convergence accuracy gradually becomes close to the optimal solution.

The convergence curves of the ESGA and the comparison algorithms on the CEC2022 test set are given in Figure 5. The ESGA shows strong a convergence speed on the unimodal function F1, achieving the best convergence accuracy. For the basic function, the ESGA demonstrates excellent search capability, achieving better fitness values with fewer iterations. For hybrid and combinatorial functions, the ESGA achieves faster convergence and excellent solution quality on most functions. Overall, the ESGA effectively avoids stagnation at local optima and premature convergence, and shows better convergence speed and convergence accuracy, which outperforms the original SGA and other comparative algorithms. These results further confirm the effectiveness of the proposed algorithm.

Additionally, we performed a boxplot analysis to investigate the distribution characteristics of solutions obtained using the ESGA compared to other competing algorithms on the challenging CEC2022 benchmark suite (as depicted in Figure 6). This analysis clearly demonstrates that our proposed method surpasses its competitors across most functions in terms of performance.

Table 6 presents the values of the 12 CEC2022 test functions calculated using the Wilcoxon rank sum test. Two independent samples (ESGA vs. SGA, MRFO, DBO, EO, RIME, GLS-MPA, EOSMA, AFDB-ARO, and LSHADE) are compared and it is determined whether the proposed algorithm is statistically different from the others. In the table, the symbol “+” indicates that the proposed algorithm outperforms the compared algorithms, the symbol “−” indicates a worse performance relative to the algorithm, and the symbol “=” indicates that both performances are similar. Table 6 shows that the proposed ESGA obtains more “+”. The results are as follows: 24/0/0, 23/1/0, 19/5/0, 19/5/0, 20/4/0, 18/5/1, 20/3/1, 19/3/2, and 21/2/1. Specifically, the ESGA dominates across the board when compared to these highly cited basic algorithms and is not weaker on any function. When compared to the improved algorithms, the ESGA is only weaker on individual functions. This indicates that the ESGA exhibits a statistically significant performance advantage over the comparison algorithms on most of the benchmark functions.

**Figure 7:**
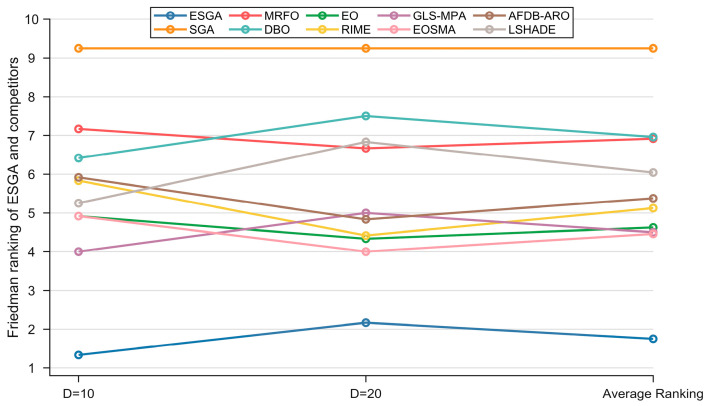
The Friedman ranking of ESGA and competitors on CEC2022 test suite.

Table 7 summarizes the Friedman test results of the ESGA and comparison algorithms on the CEC2022 test suite, with the rankings shown in Figure 7. The significance level of the Friedman test is 0.05. According to Table 7, the p-values for both 10D and 20D are less than 0.05, indicating significant differences between these comparison algorithms and the ESGA. The ESGA achieves Friedman scores of 1.333 and 2.167 for 10D and 20D, respectively, compared to the original SGA’s scores of 9.250 and 9.250, respectively, which underscores the large performance gap between the ESGA and the basic SGA. In addition, as shown in Figure 7, the ESGA has the least fluctuation in the Friedman rankings, suggesting that it is insensitive to dimensionality changes and therefore has better scalability.

**Figure 8:**
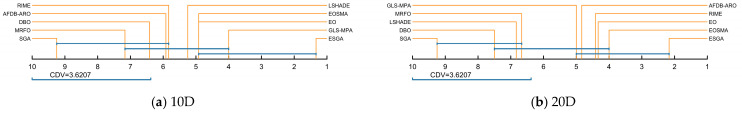
The visualization of Nemenyi test results of ESGA and competitors on CEC2022 test suite.

Based on the Friedman rankings obtained from the ESGA and comparison algorithms, the Nemenyi test was used to quantify the performance differences between these algorithms, as shown in Figure 8. In the Nemenyi test, the critical difference value (CDV) is first calculated based on the number of algorithms and the number of functions involved in the comparison. Then, the CDV is compared with the ranking difference between the two algorithms; if the CDV is greater than this interpolated value, it means that there is a difference between these two algorithms, and if vice versa, there is no difference. For 10D, the ESGA performed no difference with EOSMA, GLS-MPA, or EO and significantly outperformed LSHADE, RIME, AFDB-ARO, DBO, MRFO, and the SGA. For 20D, the performance of the ESGA is similar to that of EOSMA, RIME, EO, AFDB-ARO, and GLS-MPA, and significantly better than that of LSHADE, DBO, MRFO, and the SGA. In conclusion, the proposed ESGA has a clear advantage in comparison with these meta-heuristic algorithms, and is a promising improved algorithm.

### 5.3. Comparison Test Using Robot Path Planning

In this subsection, we evaluate the performance of the ESGA in the robot path planning problem and compare it with the top-ranked GLS-MPA and EOSMA in Section 4.2. To be fair, we set the population size to 30 and the maximum number of function evaluations to 3000. Each algorithm is run 30 times and the best value (Best), average (Avg), and standard deviation (Std) are calculated for each algorithm.

**Figure 9:**
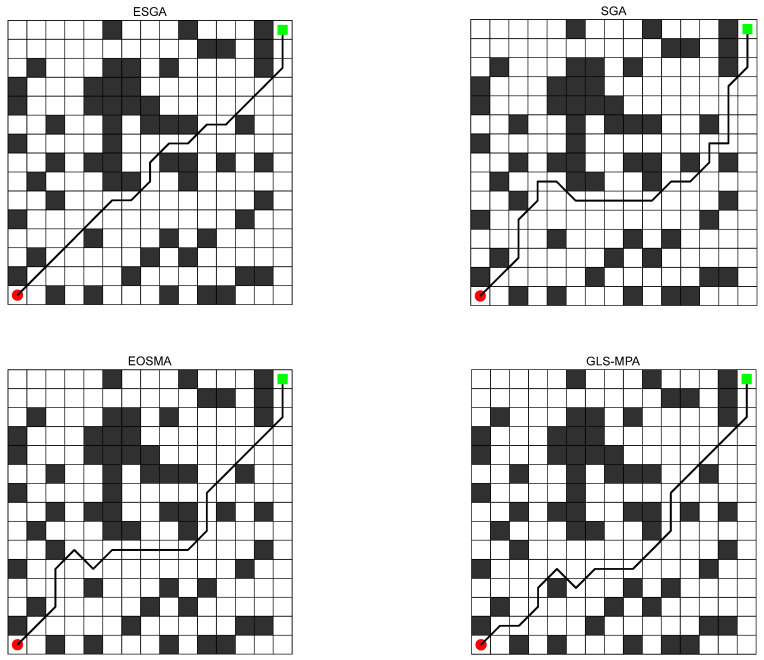
Path diagrams for ESGA and competitors.

**Figure 10:**
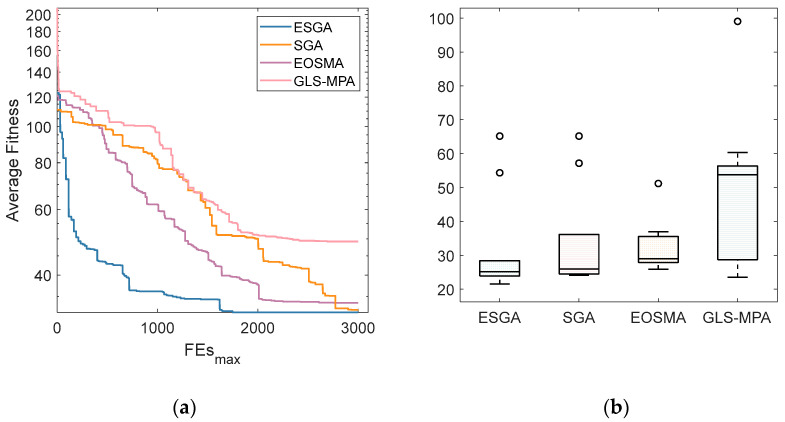
The convergence curves and boxplots of ESGA and competitors on robot path planning.

The optimal path planning graphs for solving the 15 × 15 raster graphs for the ESGA and the comparison algorithms are given in Figure 9, respectively. Green represents the starting point and red represents the ending point. Black indicates obstacles and white indicates passable. It can be seen that all four algorithms plan a feasible path, but that the ESGA gives a significantly smoother solution with a shorter path length. From Table 8, it can be learned that the ESGA proposed in this paper is the best among all the algorithms in the two metrics of optimal path length and average path length, and that the GLS-MPA algorithm is more stable, but its performance is average. The average length convergence curves of the four algorithms are plotted in Figure 10a, which shows that the ESGA has better convergence accuracy and a faster convergence speed. The boxplots are obtained based on the solutions of the four algorithms for solving the robot path planning problem, and as shown in Figure 10b, the distribution of solutions for the ESGA is denser and smaller. This is because the introduction of multiple strategies makes the search of the algorithms more comprehensive, which greatly improves the search capability of the ESGA and enables it to plan routes with lower costs.

## 6. Conclusions

Building upon improvement strategies, this paper introduces the enhanced snow geese optimizer (ESGA) as a new reference for enhancing other algorithms. The performance of the ESGA was evaluated using two sets of mathematical benchmark test suites, namely the CEC2017 and CEC2022 benchmark test functions. These tests were conducted to analyze the algorithm’s capabilities in global searches and local contractions. The results demonstrate that the ESGA exhibits strong performance in terms of convergence speed, solution accuracy, precision, and stability, outperforming other competitive algorithms and showcasing sufficient competitiveness. In order to further verify the practical application value of the ESGA, it is applied to the robot path planning problem. The optimization results of the robot path planning problem highlight the superiority of the ESGA over other optimization algorithms.

However, it is undeniable that the research level of the ESGA improvement in this paper is still relatively shallow. Due to the “no free lunch” theory, it does not show the best results on all problems. At the same time, some strategies have excellent performance when acting on certain function problems alone, but the optimization accuracy of the improved algorithm after multi-strategy fusion is reduced. How to effectively select the improvement strategy, increase the mutual gain of multiple improvement strategies in the algorithm, reduce the conflict between strategies, and use parallel computing and distributed computing technology to optimize the computational efficiency of the algorithm still needs to be further tested and explored. In the follow-up research process, further improvement research will be carried out to solve the above problems.

For future work, the improvement strategy of the ESGA can be used as a reference for enhancing other algorithms, extending the ESGA to a multi-objective algorithm for solving various multi-objective optimization problems, such as multi-UAV path problems. Additionally, the ESGA can be extended and applied to solving optimization problems in different fields and various real-world engineering applications, such as neural network hyper-parameter optimization.

## Figures and Tables

**Table 1:** Parameter settings of ESGA and other competing algorithms.

Algorithm	Parameter Settings
ESGA	No parameter
SGA	No parameter
MRFO	S=2
DBO	$L=0.8,P=0.11,arc=1.5,s=0.5,N_{m}=4,M=5$
EO	$a_{1}=2,a_{2}=1,GP=0.5$
RIME	W=5
GLS-MPA	$F=0.2,P=0.5,A=30,C_{m}=2500$
EOSMA	GP=0.5,z=0.6,q=0.2
AFDB-ARO	$F=0.2,P=0.5,A=30,C_{m}=2500$
LSHADE	$F=0.5,Cr=0.5,p=0{.11,N}_{\min}=4$

**Table 2:** Descriptions of SGA variants with different strategies.

Strategy	SGA	SGA-AS	SGA-DG	SGA-DS	SGA-ASDG	SGA-ASDS	SGA-DGDS	ESGA
ASS	N	Y	N	N	Y	Y	N	Y
DGS	N	N	Y	N	Y	N	Y	Y
DSS	N	N	N	Y	N	Y	Y	Y

**Table 3:** Friedman test results of SGA variants with different strategies on CEC2017 test suite.

Algorithm	SGA	SGA-AS	SGA-DG	SGA-DS	SGA-ASDG	SGA-ASDS	SGA-DGDS	ESGA	*p*-Value
10D	7.897	6.724	4.000	5.517	2.793	5.103	2.483	1.483	2.35 × 10^−32^
30D	7.931	6.724	4.103	5.690	3.069	4.862	2.483	1.138	5.20 × 10^−34^
50D	7.828	6.552	4.517	5.828	3.034	4.828	2.276	1.138	1.03 × 10^−33^
100D	7.793	6.966	4.552	5.552	3.034	4.483	2.621	1.000	3.93 × 10^−34^
Average ranking	7.862	6.741	4.293	5.647	2.983	4.819	2.466	1.190	

**Table 4:** Qualitative results of ESGA and competitors on CEC2022 test suite (10D).

Function No.	Indicators	ESGA	SGA	MRFO	DBO	EO	RIME	GLS-MPA	EOSMA	AFDB-ARO	LSHADE
F1	Min	3.000 × 10^2^	3.070 × 10^2^	5.305 × 10^2^	8.887 × 10^2^	5.782 × 10^2^	3.451 × 10^2^	3.200 × 10^2^	4.084 × 10^2^	1.871 × 10^3^	3.082 × 10^2^
	Avg	3.000 × 10^2^	3.392 × 10^3^	1.386 × 10^3^	3.389 × 10^3^	1.911 × 10^3^	5.214 × 10^2^	6.268 × 10^2^	1.006 × 10^3^	5.840 × 10^3^	3.375 × 10^2^
	Std	7.029 × 10^−4^	2.138 × 10^3^	6.118 × 10^2^	1.749 × 10^3^	1.204 × 10^3^	1.705 × 10^2^	2.678 × 10^2^	3.938 × 10^2^	1.664 × 10^3^	2.625 × 10^1^
F2	Min	4.000 × 10^2^	4.071 × 10^2^	4.007 × 10^2^	4.006 × 10^2^	4.049 × 10^2^	4.006 × 10^2^	4.005 × 10^2^	4.012 × 10^2^	4.071 × 10^2^	4.055 × 10^2^
	Avg	4.063 × 10^2^	4.776 × 10^2^	4.167 × 10^2^	4.192 × 10^2^	4.106 × 10^2^	4.179 × 10^2^	4.105 × 10^2^	4.091 × 10^2^	4.344 × 10^2^	4.075 × 10^2^
	Std	3.074	1.074 × 10^2^	2.188 × 10^1^	2.443 × 10^1^	5.795	2.443 × 10^1^	1.498 × 10^1^	7.239	2.319 × 10^1^	1.850
F3	Min	6.002 × 10^2^	6.052 × 10^2^	6.009 × 10^2^	6.000 × 10^2^	6.005 × 10^2^	6.003 × 10^2^	6.004 × 10^2^	6.006 × 10^2^	6.001 × 10^2^	6.012 × 10^2^
	Avg	6.017 × 10^2^	6.223 × 10^2^	6.030 × 10^2^	6.022 × 10^2^	6.014 × 10^2^	6.018 × 10^2^	6.047 × 10^2^	6.015 × 10^2^	6.018 × 10^2^	6.030 × 10^2^
	Std	1.559	1.118 × 10^1^	1.133	3.149	6.195 × 10^−1^	1.180	3.299	5.912 × 10^−1^	1.643	1.012
F4	Min	8.040 × 10^2^	8.087 × 10^2^	8.119 × 10^2^	8.110 × 10^2^	8.071 × 10^2^	8.112 × 10^2^	8.096 × 10^2^	8.101 × 10^2^	8.173 × 10^2^	8.164 × 10^2^
	Avg	8.133 × 10^2^	8.286 × 10^2^	8.204 × 10^2^	8.325 × 10^2^	8.151 × 10^2^	8.211 × 10^2^	8.176 × 10^2^	8.191 × 10^2^	8.298 × 10^2^	8.392 × 10^2^
	Std	4.648	1.318 × 10^1^	7.453	1.266 × 10^1^	4.274	7.373	4.921	5.345	6.382	8.848
F5	Min	9.000 × 10^2^	9.321 × 10^2^	9.004 × 10^2^	9.001 × 10^2^	9.002 × 10^2^	9.004 × 10^2^	9.031 × 10^2^	9.003 × 10^2^	9.053 × 10^2^	9.017 × 10^2^
	Avg	9.014 × 10^2^	1.150 × 10^3^	9.192 × 10^2^	9.411 × 10^2^	9.026 × 10^2^	9.065 × 10^2^	9.289 × 10^2^	9.017 × 10^2^	9.332 × 10^2^	9.052 × 10^2^
	Std	1.963	1.836 × 10^2^	3.745 × 10^1^	7.505 × 10^1^	2.545	7.311	1.850 × 10^1^	1.296	1.561 × 10^1^	3.665
F6	Min	1.823 × 10^3^	1.918 × 10^3^	3.286 × 10^3^	1.854 × 10^3^	2.467 × 10^3^	1.961 × 10^3^	1.927 × 10^3^	4.052 × 10^3^	1.938 × 10^3^	1.967 × 10^3^
	Avg	2.310 × 10^3^	4.313 × 10^3^	1.625 × 10^4^	4.727 × 10^3^	5.655 × 10^3^	6.930 × 10^3^	4.112 × 10^3^	1.415 × 10^4^	2.723 × 10^3^	2.780 × 10^3^
	Std	1.523 × 10^3^	2.136 × 10^3^	1.116 × 10^4^	2.430 × 10^3^	3.213 × 10^3^	5.722 × 10^3^	1.823 × 10^3^	1.026 × 10^4^	7.638 × 10^2^	8.892 × 10^2^
F7	Min	2.005 × 10^3^	2.022 × 10^3^	2.019 × 10^3^	2.020 × 10^3^	2.019 × 10^3^	2.020 × 10^3^	2.015 × 10^3^	2.014 × 10^3^	2.013 × 10^3^	2.033 × 10^3^
	Avg	2.024 × 10^3^	2.053 × 10^3^	2.035 × 10^3^	2.025 × 10^3^	2.028 × 10^3^	2.025 × 10^3^	2.024 × 10^3^	2.029 × 10^3^	2.021 × 10^3^	2.042 × 10^3^
	Std	8.270	2.089 × 10^1^	7.063	6.855	4.085	2.417	4.570	5.459	2.260	4.909
F8	Min	2.201 × 10^3^	2.223 × 10^3^	2.213 × 10^3^	2.217 × 10^3^	2.211 × 10^3^	2.208 × 10^3^	2.208 × 10^3^	2.213 × 10^3^	2.208 × 10^3^	2.219 × 10^3^
	Avg	2.221 × 10^3^	2.240 × 10^3^	2.229 × 10^3^	2.226 × 10^3^	2.227 × 10^3^	2.223 × 10^3^	2.222 × 10^3^	2.227 × 10^3^	2.221 × 10^3^	2.229 × 10^3^
	Std	7.912	3.216 × 10^1^	3.878	6.005	4.715	4.884	4.170	4.311	3.269	3.454
F9	Min	2.529 × 10^3^	2.531 × 10^3^	2.535 × 10^3^	2.529 × 10^3^	2.529 × 10^3^	2.529 × 10^3^	2.529 × 10^3^	2.530 × 10^3^	2.531 × 10^3^	2.529 × 10^3^
	Avg	2.529 × 10^3^	2.609 × 10^3^	2.543 × 10^3^	2.536 × 10^3^	2.533 × 10^3^	2.530 × 10^3^	2.530 × 10^3^	2.532 × 10^3^	2.537 × 10^3^	2.530 × 10^3^
	Std	4.266 × 10^−6^	4.869 × 10^1^	1.075 × 10^1^	2.070 × 10^1^	3.982	7.675 × 10^−1^	2.613 × 10^−1^	1.723	1.206 × 10^1^	3.058 × 10^−1^
F10	Min	2.500 × 10^3^	2.500 × 10^3^	2.500 × 10^3^	2.500 × 10^3^	2.500 × 10^3^	2.500 × 10^3^	2.500 × 10^3^	2.500 × 10^3^	2.501 × 10^3^	2.500 × 10^3^
	Avg	2.500 × 10^3^	2.519 × 10^3^	2.505 × 10^3^	2.505 × 10^3^	2.504 × 10^3^	2.516 × 10^3^	2.501 × 10^3^	2.500 × 10^3^	2.501 × 10^3^	2.501 × 10^3^
	Std	9.063 × 10^−2^	4.564 × 10^1^	2.156 × 10^1^	2.046 × 10^1^	2.070 × 10^1^	4.055 × 10^1^	1.060 × 10^−1^	9.232 × 10^−2^	3.713 × 10^−1^	1.455 × 10^−1^
F11	Min	2.600 × 10^3^	2.747 × 10^3^	2.639 × 10^3^	2.615 × 10^3^	2.685 × 10^3^	2.671 × 10^3^	2.719 × 10^3^	2.718 × 10^3^	2.756 × 10^3^	2.754 × 10^3^
	Avg	2.657 × 10^3^	3.111 × 10^3^	2.780 × 10^3^	2.765 × 10^3^	2.915 × 10^3^	3.043 × 10^3^	2.771 × 10^3^	2.863 × 10^3^	2.816 × 10^3^	2.902 × 10^3^
	Std	1.113 × 10^2^	2.998 × 10^2^	1.394 × 10^2^	1.296 × 10^2^	1.597 × 10^2^	1.728 × 10^2^	1.916 × 10^1^	1.393 × 10^2^	4.159 × 10^1^	1.422 × 10^2^
F12	Min	2.860 × 10^3^	2.864 × 10^3^	2.866 × 10^3^	2.862 × 10^3^	2.862 × 10^3^	2.862 × 10^3^	2.861 × 10^3^	2.863 × 10^3^	2.868 × 10^3^	2.861 × 10^3^
	Avg	2.863 × 10^3^	2.878 × 10^3^	2.873 × 10^3^	2.865 × 10^3^	2.864 × 10^3^	2.865 × 10^3^	2.864 × 10^3^	2.865 × 10^3^	2.874 × 10^3^	2.864 × 10^3^
	Std	1.405	1.806 × 10^1^	8.931	1.999	1.150	2.214	1.319	5.491 × 10^−1^	2.982	9.318 × 10^−1^

**Table 5:** Qualitative results of ESGA and competitors on CEC2022 test suite (20D).

Function No.	Indicators	ESGA	SGA	MRFO	DBO	EO	RIME	GLS-MPA	EOSMA	AFDB-ARO	LSHADE
F1	Min	3.000 × 10^2^	8.507 × 10^3^	8.784 × 10^3^	2.124 × 10^4^	1.236 × 10^4^	4.966 × 10^3^	3.331 × 10^3^	5.645 × 10^3^	1.956 × 10^4^	9.058 × 10^3^
	Avg	3.018 × 10^2^	2.848 × 10^4^	1.980 × 10^4^	3.545 × 10^4^	2.471 × 10^4^	1.426 × 10^4^	9.735 × 10^3^	1.433 × 10^4^	2.959 × 10^4^	1.615 × 10^4^
	Std	3.173	2.217 × 10^4^	7.099 × 10^3^	8.568 × 10^3^	8.166 × 10^3^	5.125 × 10^3^	3.136 × 10^3^	4.159 × 10^3^	5.669 × 10^3^	5.017 × 10^3^
F2	Min	4.339 × 10^2^	4.827 × 10^2^	4.688 × 10^2^	4.503 × 10^2^	4.639 × 10^2^	4.472 × 10^2^	4.857 × 10^2^	4.702 × 10^2^	4.779 × 10^2^	4.858 × 10^2^
	Avg	4.591 × 10^2^	6.565 × 10^2^	5.324 × 10^2^	5.256 × 10^2^	5.092 × 10^2^	4.847 × 10^2^	5.525 × 10^2^	5.175 × 10^2^	5.681 × 10^2^	5.090 × 10^2^
	Std	2.314 × 10^1^	1.281 × 10^2^	4.543 × 10^1^	6.388 × 10^1^	2.756 × 10^1^	3.958 × 10^1^	5.234 × 10^1^	2.601 × 10^1^	5.195 × 10^1^	1.481 × 10^1^
F3	Min	6.043 × 10^2^	6.283 × 10^2^	6.110 × 10^2^	6.111 × 10^2^	6.044 × 10^2^	6.045 × 10^2^	6.120 × 10^2^	6.053 × 10^2^	6.014 × 10^2^	6.157 × 10^2^
	Avg	6.097 × 10^2^	6.477 × 10^2^	6.201 × 10^2^	6.231 × 10^2^	6.088 × 10^2^	6.095 × 10^2^	6.221 × 10^2^	6.092 × 10^2^	6.092 × 10^2^	6.216 × 10^2^
	Std	3.426	1.186 × 10^1^	6.920	8.136	1.845	3.255	5.900	2.558	4.739	2.532
F4	Min	8.189 × 10^2^	8.445 × 10^2^	8.650 × 10^2^	8.743 × 10^2^	8.497 × 10^2^	8.453 × 10^2^	8.379 × 10^2^	8.479 × 10^2^	8.715 × 10^2^	9.178 × 10^2^
	Avg	8.415 × 10^2^	9.023 × 10^2^	8.961 × 10^2^	9.244 × 10^2^	8.801 × 10^2^	8.759 × 10^2^	8.827 × 10^2^	8.844 × 10^2^	9.027 × 10^2^	9.402 × 10^2^
	Std	1.471 × 10^1^	2.520 × 10^1^	2.191 × 10^1^	2.625 × 10^1^	1.227 × 10^1^	1.900 × 10^1^	2.326 × 10^1^	1.394 × 10^1^	1.714 × 10^1^	9.359
F5	Min	9.276 × 10^2^	1.339 × 10^3^	1.010 × 10^3^	1.517 × 10^3^	9.275 × 10^2^	9.626 × 10^2^	1.104 × 10^3^	9.278 × 10^2^	1.184 × 10^3^	1.182 × 10^3^
	Avg	1.009 × 10^3^	2.314 × 10^3^	1.722 × 10^3^	2.340 × 10^3^	1.060 × 10^3^	1.466 × 10^3^	1.431 × 10^3^	1.009 × 10^3^	1.559 × 10^3^	1.409 × 10^3^
	Std	9.871 × 10^1^	5.110 × 10^2^	6.378 × 10^2^	6.309 × 10^2^	9.279 × 10^1^	6.769 × 10^2^	2.206 × 10^2^	4.949 × 10^1^	1.928 × 10^2^	1.356 × 10^2^
F6	Min	2.030 × 10^3^	4.193 × 10^3^	1.590 × 10^5^	2.311 × 10^3^	2.177 × 10^4^	1.950 × 10^4^	3.438 × 10^3^	6.569 × 10^4^	2.128 × 10^4^	1.145 × 10^7^
	Avg	8.505 × 10^3^	1.870 × 10^7^	7.970 × 10^5^	1.136 × 10^6^	3.418 × 10^5^	8.791 × 10^5^	9.867 × 10^5^	5.427 × 10^5^	8.254 × 10^4^	1.998 × 10^7^
	Std	6.488 × 10^3^	9.124 × 10^7^	4.826 × 10^5^	5.347 × 10^6^	4.088 × 10^5^	8.182 × 10^5^	1.484 × 10^6^	5.766 × 10^5^	5.492 × 10^4^	7.041 × 10^6^
F7	Min	2.042 × 10^3^	2.071 × 10^3^	2.066 × 10^3^	2.043 × 10^3^	2.053 × 10^3^	2.046 × 10^3^	2.037 × 10^3^	2.062 × 10^3^	2.042 × 10^3^	2.115 × 10^3^
	Avg	2.075 × 10^3^	2.155 × 10^3^	2.115 × 10^3^	2.107 × 10^3^	2.092 × 10^3^	2.091 × 10^3^	2.081 × 10^3^	2.098 × 10^3^	2.088 × 10^3^	2.163 × 10^3^
	Std	1.853 × 10^1^	4.455 × 10^1^	3.639 × 10^1^	3.628 × 10^1^	2.231 × 10^1^	2.633 × 10^1^	2.515 × 10^1^	2.221 × 10^1^	3.155 × 10^1^	2.599 × 10^1^
F8	Min	2.222 × 10^3^	2.231 × 10^3^	2.232 × 10^3^	2.228 × 10^3^	2.229 × 10^3^	2.227 × 10^3^	2.226 × 10^3^	2.233 × 10^3^	2.224 × 10^3^	2.244 × 10^3^
	Avg	2.230 × 10^3^	2.326 × 10^3^	2.250 × 10^3^	2.273 × 10^3^	2.241 × 10^3^	2.240 × 10^3^	2.231 × 10^3^	2.240 × 10^3^	2.235 × 10^3^	2.264 × 10^3^
	Std	6.356	1.016 × 10^2^	2.997 × 10^1^	5.373 × 10^1^	2.324 × 10^1^	2.338 × 10^1^	4.586	3.342	7.858	1.002 × 10^1^
F9	Min	2.481 × 10^3^	2.522 × 10^3^	2.500 × 10^3^	2.481 × 10^3^	2.486 × 10^3^	2.484 × 10^3^	2.487 × 10^3^	2.488 × 10^3^	2.484 × 10^3^	2.491 × 10^3^
	Avg	2.482 × 10^3^	2.611 × 10^3^	2.514 × 10^3^	2.512 × 10^3^	2.501 × 10^3^	2.496 × 10^3^	2.513 × 10^3^	2.500 × 10^3^	2.489 × 10^3^	2.501 × 10^3^
	Std	3.217	5.949 × 10^1^	1.131 × 10^1^	2.430 × 10^1^	1.250 × 10^1^	1.672 × 10^1^	1.944 × 10^1^	6.073	4.283	5.025
F10	Min	2.500 × 10^3^	2.501 × 10^3^	2.501 × 10^3^	2.501 × 10^3^	2.501 × 10^3^	2.501 × 10^3^	2.501 × 10^3^	2.501 × 10^3^	2.436 × 10^3^	2.501 × 10^3^
	Avg	2.519 × 10^3^	4.043 × 10^3^	2.616 × 10^3^	2.798 × 10^3^	2.927 × 10^3^	2.883 × 10^3^	2.510 × 10^3^	2.508 × 10^3^	2.514 × 10^3^	2.518 × 10^3^
	Std	5.475 × 10^1^	1.588 × 10^3^	4.473 × 10^2^	7.239 × 10^2^	1.071 × 10^3^	5.248 × 10^2^	3.359 × 10^1^	3.610 × 10^1^	3.864 × 10^1^	5.612 × 10^1^
F11	Min	2.902 × 10^3^	3.740 × 10^3^	3.279 × 10^3^	3.447 × 10^3^	3.474 × 10^3^	4.036 × 10^3^	3.847 × 10^3^	3.740 × 10^3^	2.949 × 10^3^	5.395 × 10^3^
	Avg	3.010 × 10^3^	5.386 × 10^3^	3.587 × 10^3^	4.556 × 10^3^	4.226 × 10^3^	4.570 × 10^3^	5.130 × 10^3^	4.213 × 10^3^	3.299 × 10^3^	7.868 × 10^3^
	Std	1.406 × 10^2^	8.346 × 10^2^	1.952 × 10^2^	9.359 × 10^2^	3.311 × 10^2^	3.443 × 10^2^	6.091 × 10^2^	2.878 × 10^2^	1.082 × 10^2^	1.017 × 10^3^
F12	Min	2.943 × 10^3^	2.980 × 10^3^	3.006 × 10^3^	2.944 × 10^3^	2.950 × 10^3^	2.944 × 10^3^	2.945 × 10^3^	2.968 × 10^3^	2.988 × 10^3^	2.970 × 10^3^
	Avg	2.983 × 10^3^	3.102 × 10^3^	3.076 × 10^3^	2.969 × 10^3^	2.967 × 10^3^	2.971 × 10^3^	2.979 × 10^3^	2.996 × 10^3^	3.056 × 10^3^	2.981 × 10^3^
	Std	3.126 × 10^1^	1.109 × 10^2^	3.611 × 10^1^	2.934 × 10^1^	1.156 × 10^1^	1.660 × 10^1^	2.264 × 10^1^	1.516 × 10^1^	4.014 × 10^1^	7.135

**Table 6:** Wilcoxon rank sum test results of ESGA and competitors on CEC2022 test suite.

ESGA vs. +/=/−	SGA	MRFO	DBO	EO	RIME	GLS-MPA	EOSMA	AFDB-ARO	LSHADE
10D	12/0/0	11/1/0	9/3/0	9/3/0	10/2/0	9/3/0	11/1/0	10/1/1	11/1/0
20D	12/0/0	12/0/0	10/2/0	10/2/0	10/2/0	9/2/1	9/2/1	9/2/1	10/1/1
Total	24/0/0	23/1/0	19/5/0	19/5/0	20/4/0	18/5/1	20/3/1	19/3/2	21/2/1

**Table 7:** Friedman test results of ESGA and competitors on CEC2022 test suite.

Algorithm	10D	20D	Average Ranking
ESGA	1.333	2.167	1.750
SGA	9.250	9.250	9.250
MRFO	7.167	6.667	6.917
DBO	6.417	7.500	6.958
EO	4.917	4.333	4.625
RIME	5.833	4.417	5.125
GLS-MPA	4.000	5.000	4.500
EOSMA	4.917	4.000	4.458
AFDB-ARO	5.917	4.833	5.375
LSHADE	5.250	6.833	6.042
*p*-value	1.00 × 10^−7^	1.35 × 10^−7^	

**Table 8:** Qualitative results of ESGA and competitors on robot path planning.

Index	ESGA	SGA	EOSMA	GLS-MPA
Best	21.56	25.90	24.14	23.56
Avg	31.78	32.26	33.70	49.18
Std	15.12	7.65	15.03	22.59

## Data Availability

The data is provided within the manuscript.
